# Change in potato consumption among Norwegian women 1998-2005—The Norwegian Women and Cancer study (NOWAC)

**DOI:** 10.1371/journal.pone.0179441

**Published:** 2017-06-09

**Authors:** Ambrose Ojodale Attah, Tonje Braaten, Guri Skeie

**Affiliations:** Department of Community Medicine, UiT – The Arctic University of Norway, Tromsø, Norway; Medical University of Vienna, AUSTRIA

## Abstract

Studies have shown that potato consumption in Norway have been on the decline in recent years. Increase in income and the association of potato consumption with weight gain and chronic diseases like type 2 diabetes have been identified as some of the factors responsible for the change. The aim of this study was to describe the change in potato consumption within persons and how non-dietary variables influenced that change among participants in the Norwegian Women and Cancer study (NOWAC). A prospective analysis was performed in the NOWAC cohort using linear regression. Data on dietary, lifestyle, socioeconomic and health-related factors were collected by mailed questionnaires. The change in potato consumption among 38,820 women aged 41–70 years was investigated using two measurements taken at intervals of 4–6 years. At baseline, mean intake was 112g per day; this had decreased to 94.5g per day at the second measurement. Results showed that the percentage of women who reported that they ate less than 1 potato a day increased from 24.6% at baseline to 35.5% at the second measurement. Those who reported that they ate more than 3 potatoes a day had decreased from 20.2% of the participants at baseline to 12.1% at the second measurement. Multivariable adjusted results show that geography was an important predictor of potato consumption at second measurement. Living in the north compared to Oslo (the capital) was associated with higher intake of potato at second measurement (B: 0.60, 95% CI: 0.55–0.65). Compared to women living with a partner, living alone was associated with lower potato intake at second measurement (B: -0.13, 95% CI: -0.17 –-0.09) while living with children tended to be associated with higher potato intake at second measurement (B: 0.01, 95% CI: -0.02–0.04). Younger age, more years of education, higher income or BMI was associated with a lower potato intake at second measurement. Smoking was associated with a higher intake of potato at second measurement (B: 0.03, 95% CI: 0.00–0.06 for smokers compared to non-smokers). Having diabetes at baseline was associated with lower intake of potato at second measurement (B: -0.04, 95% CI: -0.14 –-0.06 for non-diabetics compared to diabetics). Potato consumption among women in the NOWAC study showed a decline over the period studied. Change in the consumption was found to be influenced by age, education, income, household structure, region of residence as well as health-related factors like smoking and diabetes. The use of repeated measures is necessary to continue the monitoring and also to understand the stability and direction of the possible change in diet of a population.

## Introduction

Potato is the third most important food crop in the world after rice and wheat in terms of human consumption. More than a billion people worldwide eat potato, and global total crop production exceeds 300 million metric tons [1] making it a staple in the diets of many people [2]. In addition to being easy to grow on small plots, cheap to purchase, and ready to cook without expensive processing, potatoes are energy-rich and nutritious [1].

Potatoes contribute nutrients like vitamin C, folate and dietary fibre to the diet [3]. While high intake of dietary fibre is associated with a reduced risk of diverticular disease [4], preparations from potato dietary fibre have been shown to abolish the negative impacts of acrylamide on the histologic structure, regeneration, and innervation of the small intestinal wall and the absorptive function of the small intestinal mucosa [5]. Potato is also an important source of kynurenic acid which is believed to possess neuroprotective, anti-inflammatory, antioxidant and antiproliferative properties [6].

Potato consumption has been associated with favourable effects on cardiometabolic health [7] but the high glycemic index [8], association with higher weight gain and risk of type 2 diabetes mellitus [9, 10] and its association with an increased risk of hypertension [11] seem to discourage its consumption. On the contrary, other studies have reported that potato consumption does not promote obesity [12], and that the glycemic index of potato vary depending on variety [13], where it is grown [14], the preparation methods [13], and the contents of the potato meal [15]. Still, long-term health effects of potato consumption have not been much studied, and therefore no specific advice on potato consumption was given in food based dietary guidelines issued in Norway in 2011 [16].

Cross-sectional studies have indicated a decrease in both the consumption and cultivation of potatoes in Norway over the years [17, 18]. The aim of this study is to examine the changes in potato consumption within persons and trends by non-dietary variables among participants in the Norwegian Women and Cancer study (NOWAC).

## Methods

### The NOWAC study

The NOWAC study is a national, population-based prospective cohort study initiated in 1991 [19]. The study is based on sampling from the national population register of Norway to ensure representativeness and adequate external validity to estimate relative risks and population attributable fractions [20]. The selected women received letters of invitation together with the questionnaire. The cohort includes 172,478 women aged 30–70 years at recruitment with repeated collection of information after 4–6 years (2 or 3 measurements including baseline) [19, 21]. Details of the NOWAC study, its scientific rationale, design, and baseline characteristics have been published elsewhere [19, 20].

Diet was assessed using a semi-quantitative food frequency questionnaire (FFQ) which contains detailed questions on dietary habits. The participants were asked to record how often they consumed more than 90 different foodstuffs during the preceding year [21]. Information about portion size was asked for some foods. The weights and portions used are mostly derived from the Norwegian weights and measures table [3]. The NOWAC FFQ has been thoroughly validated by 24h dietary recalls [22], a test-retest study [23] along with a study of how to handle missing values in dietary intake calculations [24], and against serum phospholipid fatty acid composition as biomarkers of fatty fish consumption [25]. The validation studies showed that the NOWAC FFQ has good ability to rank subjects according to food eaten frequently, and macronutrients expressed as percentage of energy intake [22].

Written informed consent was obtained from each participant, and ethical approval for the study was obtained from the Regional Ethical Committee of North-Norway and the Norwegian Data Inspectorate.

### Study sample

A total of 93,816 participants were available for inclusion in the present study based on the availability of potato information in their baseline questionnaires. The questionnaires used as baseline for this study were those completed by participants during the period 1996–1998 (66,781 women). The questionnaires used as second measurement for this study were the follow-up questionnaires completed by the same participants during the period 2002–2005 (48,767 women). The baseline measurement was a mix of first and second questionnaires while the second measurement was a mix of second and third questionnaires.

**Fig 1:**
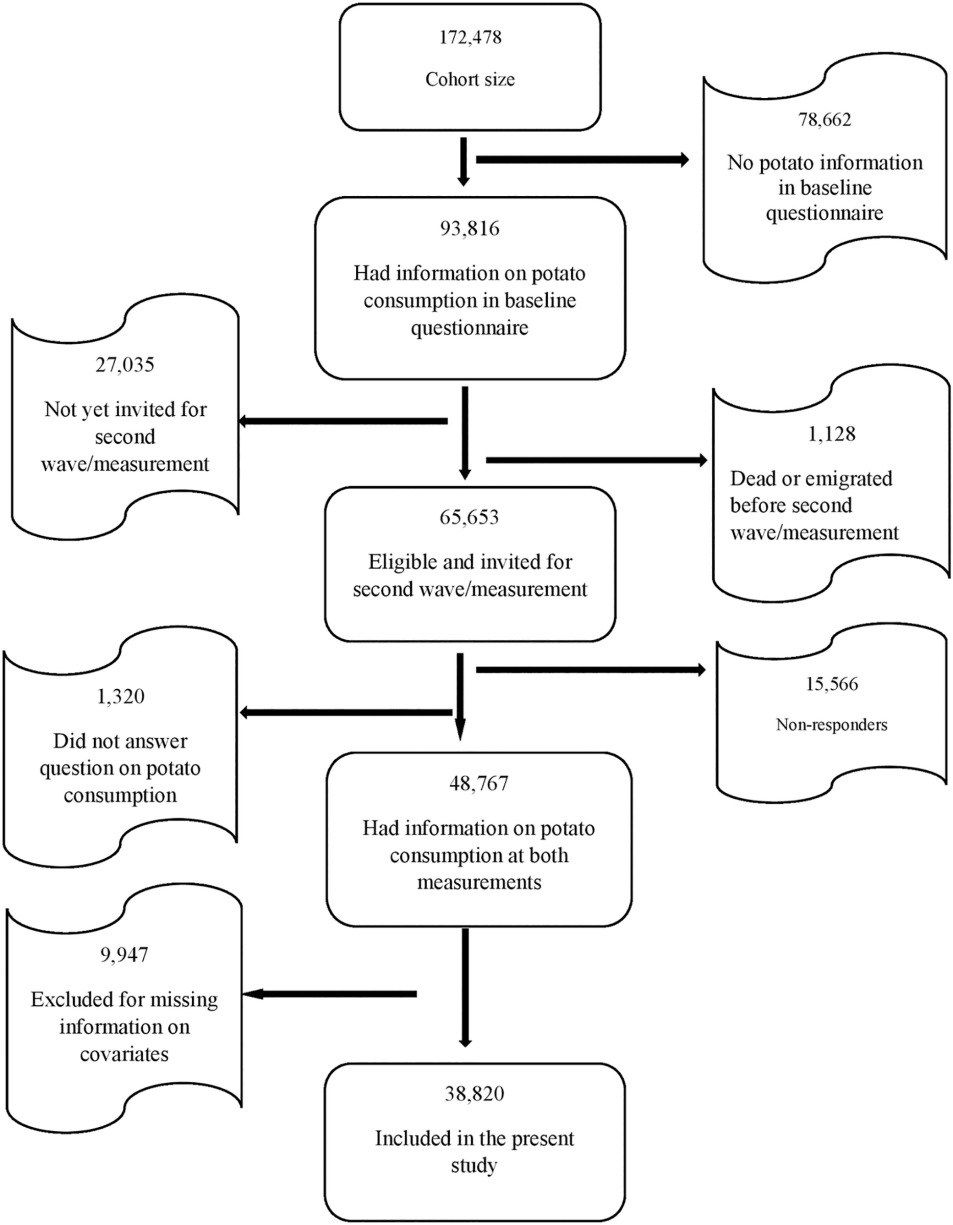
Flow chart of inclusion in the study.

We excluded 45,049 participants who had no information on potato consumption in the follow-up questionnaire. They were either dead or emigrated, yet to be invited for second wave/measurement, did not answer the question on potato consumption in the second measurement or did not respond to the invitation for second wave/measurement (Fig 1). Also, further exclusion of 9,947 participants was made due to baseline missing information on selected covariates; years of education, smoking status, level of physical activity, BMI, gross household income, marital status. Hence, 38, 820 women were finally included in the present analyses.

The participants were categorized into three potato consumption groups. Those categorized in the low potato consumption group were women who answered that they ‘seldom/never’ ate potatoes, or ate less than 1 potato a day. The moderate potato consumption group was women who answered that they ate ‘1’ or ‘2’ potatoes per day, and the high potato consumption group was women who answered that they ate ‘3’ or ‘4+’ potatoes per day. A new variable ‘change in potato consumption’ (categorized into increase, stable and decrease) was generated by subtracting the variable ‘potato consumption category’ at baseline from that at the second measurement. In order to determine whether there were children in the household, a new variable called ‘household structure’ was generated by combining the existing variables; ‘household size’ and ‘marital status’. Women who were married/living with a partner, and reported no more than two people in the household were categorized as ‘living with partner’ (no children in household). Women who were single, widowed or divorced and reported one person in the household were categorized as ‘living alone’. Women categorized as ‘living with children’ were the single, widowed or divorced who reported at least two persons in the household, and the women who answered that they were married or cohabiting and reported more than two persons in the household. Participants of the NOWAC study were asked to rate their overall physical activity level on a 10-category scale (1 being a ‘very low’ and 10 being a ‘very high’ physical activity level). We recoded this into three categories: low (1–3), moderate (4–7) and high (8–10) [26]. The body weight, height and diabetes were all self-reported. According to results from a validation study of self-reported diabetes in the NOWAC study [21], we recoded those with missing information on the question about diabetes as not having diabetes. The validation of self-reported questions on physical activity [26] and BMI [27] showed that they provide a valid ranking of physical activity and BMI for middle-aged Norwegian women.

### Statistical analysis

The statistical analyses were performed with IBM SPSS version 21. The association between the independent variables and potato consumption or change in potato consumption was evaluated using Spearman correlation coefficient where the predictor variables were ordinal and Kruskal-Wallis test where the predictor variables were categorical. Also, the distribution (%) of women in the three potato consumption groups at baseline and second measurements were evaluated by cross tabulation.

Linear regression analyses with 95% confidence intervals (CI) were performed to examine the predictors of change. The dependent variable in the linear regression analyses was ‘frequency of potato consumption at the second measurement’ i.e. not the grouped variable. The regression analyses were adjusted for potato intake at first measurement thus making the outcome variable equivalent to change in potato intake. The effect of the covariates was evaluated in age-adjusted and multivariable models, and were chosen based on previous literature on potato intake [28, 29]. The covariates include income (≤300,000; 301,000–450,000; 451,000–600,000, >600,000 Norwegian Kroner (NOK)), education (≤9, 10–12 and ≥13 years), age (40–49, 50–59, 60–70 years), region of residence (Oslo (capital), North Norway, South Norway, East Norway, West Norway, Mid Norway), smoking status (never, former, current), body mass index (BMI: weight in kilograms divided by the square of height in metres, < 25: underweight/normal weight, 25–29.9: overweight, ≥30: obese), diabetes (dichotomized), physical activity (low, moderate, high).

Tests for multicollinearity, outliers, normality, linearity and homoscedasticity in our linear regression analysis showed these assumptions were not violated. All p-values below 0.05 were considered statistically significant. All regression models were adjusted for potato intake at baseline thus providing equal estimates as for change in potato intake as the outcome.

## Results

**Fig 2:**
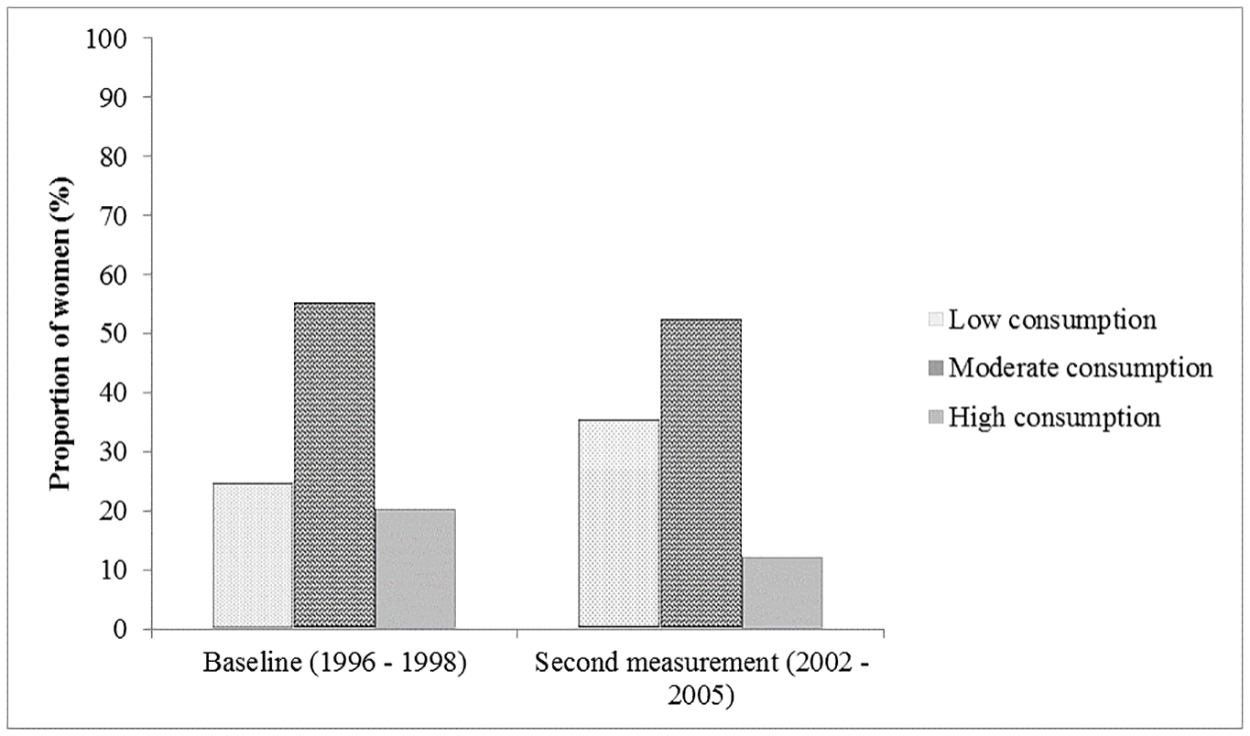
Percentage of women in the three potato consumption groups at baseline and second measurement (n = 38,820). (Low: <1 potato per day. Moderate: 1–2 potatoes per day. High: ≥ 3 potatoes per day).

Results show a change in the relative number of women in the low, moderate and high potato consumption groups between the two measurements. The percentage of women in the low potato consumption group increased from 24.6% at baseline to 35.5% at the second measurement. The percentage of those in the high consumption group decreased from 20.2% at baseline to 12.1% at the second measurement (Fig 2).

Cross tabulation shows that women who were low potato consumers at the first measurement were still predominantly (69.9%) low consumers at second measurement. The moderate consumers at the first measurement tend to stay moderate consumers, 66.1% of them were still in that group at the second measurement. Many high consumers at the first measurement were at the second measurement still high (41.4%), but a larger part (45.6%) were at second measurement moderate consumers (Table 1). On average, 112 g and 95 g of potatoes per day was consumed by each participant at the first and second measurement respectively (result not shown), a reduction of more than 15% during the period covered by this study.

**Table 1:** Potato consumption at second measurement by first measurement potato consumption group. The NOWAC study.

		**Potato Consumption at First Measurement (%)**		
**Potato Consumption at Second Measurement (%)**		Low	Moderate	High
	Low	69.9	28.3	13.0
	Moderate	27.5	66.1	45.6
	High	2.6	5.5	41.4

Low: <1 potato per day. Moderate: 1–2 potatoes per day. High: ≥ 3 potatoes per day

At baseline, the potato consumption differed significantly between the levels of all lifestyle factors. The highest percentage of the high potato consumers (43.4%) were in the north while the highest percentage of the low potato consumers were in the east of Norway (40.2%) (Table 2). Results also show that for age, household structure and smoking status, the change in potato consumption from first to second measurement differed significantly across groups (Table 3).

**Table 2:** Baseline characteristics of the study population by potato consumption group at baseline.

	Potato Consumption Groups			
Socioeconomic and health-related variables, %	Low (n = 9,555)	Moderate (n = 21,411)	High (n = 7,854)	p-value
Age (years)				
40–49	59.9	47.0	41.2	
50–59	35.9	41.6	43.4	.00^a^
60–70	4.1	11.4	15.4	
Education (years)				
≤9	14.7	26.1	38.7	
10–12	32.9	35.3	33.9	.00^a^
≥13	52.4	38.6	27.4	
Income (NOK)				
≤300,000	36.1	39.0	49.3	
301,000–450,000	24.7	31.9	31.7	.00^a^
451,000–600,000	25.0	21.1	14.5	
>600,000	14.2	7.9	4.6	
Household structure				
Living with partner	33.7	44.4	49.1	
Living alone	17.2	8.5	10.2	.00^b^
Living with children	49.1	47.2	40.6	
Region of residence				
Oslo	15.8	6.6	4.0	
East (except Oslo)	40.2	32.2	24.9	
South	5.1	3.9	4.7	.00^b^
west	18.4	22.1	15.8	
Mid	6.1	7.4	7.3	
North	14.4	27.8	43.4	
Smoking status				
never	38.1	41.3	35.9	
former	33.9	31.3	28.7	.00^b^
current	28.0	27.5	35.4	
BMI(Kg/m^2^)				
<25	66.8	61.3	60.9	
25–29.9	25.7	30.0	30.2	.00^a^
≥30	7.5	8.7	8.9	
Physical activity				
low	13.7	12.8	12.5	
moderate	73.1	74.9	72.7	.00^a^
high	13.2	12.3	14.8	
Diabetes				
Yes	1.1	1.7	1.0	
No	98.9	98.3	99.0	.83^b^

Low: <1 potato per day. Moderate: 1–2 potatoes per day. High: ≥ 3 potatoes per day.

^a^Spearman rho test for correlation with potato consumption group.

^b^Kruskal-Wallis test for significant differences in potato consumption between groups.

**Table 3:** Pattern of change observed in the consumption of potatoes among participants in the NOWAC study between baseline and second measurement according to selected baseline variables.

	Change in Potato consumption			
Socioeconomic and health-related variables, %	Decrease (n = 10,669)	Stable (n = 24,049)	Increase (n = 4,057)	p-value
Age (years)				
40–49	52.7	47.8	46.8	
50–59	38.3	41.1	43.1	.00^a^
60–70	9.0	11.1	10.1	
Education (years)				
≤ 9	25.0	26.0	27.3	
10–12	34.2	34.6	34.0	.43^a^
≥13	40.8	39.4	38.7	
Income (NOK)				
≤300,000	39.0	40.5	43.3	
301,000–450,000	31.1	29.9	28.3	
451,000–600,000	21.0	20.7	20.2	.63^a^
>600,000	9.0	8.8	8.3	
Household structure				
Living with partner	41.2	43.4	42.2	
Living alone	9.8	10.9	14.7	.00^b^
Living with children	49.0	45.7	43.1	
Region of residence				
Oslo	7.9	8.6	7.6	
East (except Oslo)	33.7	32.2	32.8	
South	4.5	4.2	5.0	.00^b^
west	19.5	20.3	19.0	
Mid	8.0	6.8	6.7	
North	26.4	27.9	29.0	
Smoking status				
never	38.3	40.2	37.6	
former	32.1	31.2	31.0	.00^b^
current	29.6	28.6	31.4	
BMI (Kg/m^2^)				
<25	62.5	62.8	62.0	
25–29.9	29.1	28.8	29.4	.43^a^
≥30	8.4	8.4	8.6	
Physical activity				
low	12.2	13.0	14.7	
moderate	74.5	74.3	71.5	.46^a^
high	13.3	12.8	13.8	
Diabetes				
Yes	1.2	1.5	1.6	
No	98.8	98.5	98.4	.23^b^

^a^Spearman rho test for correlation with change in potato consumption group.

^b^Kruskal-Wallis test for significant differences in change in potato consumption between groups.

**Fig 3:**
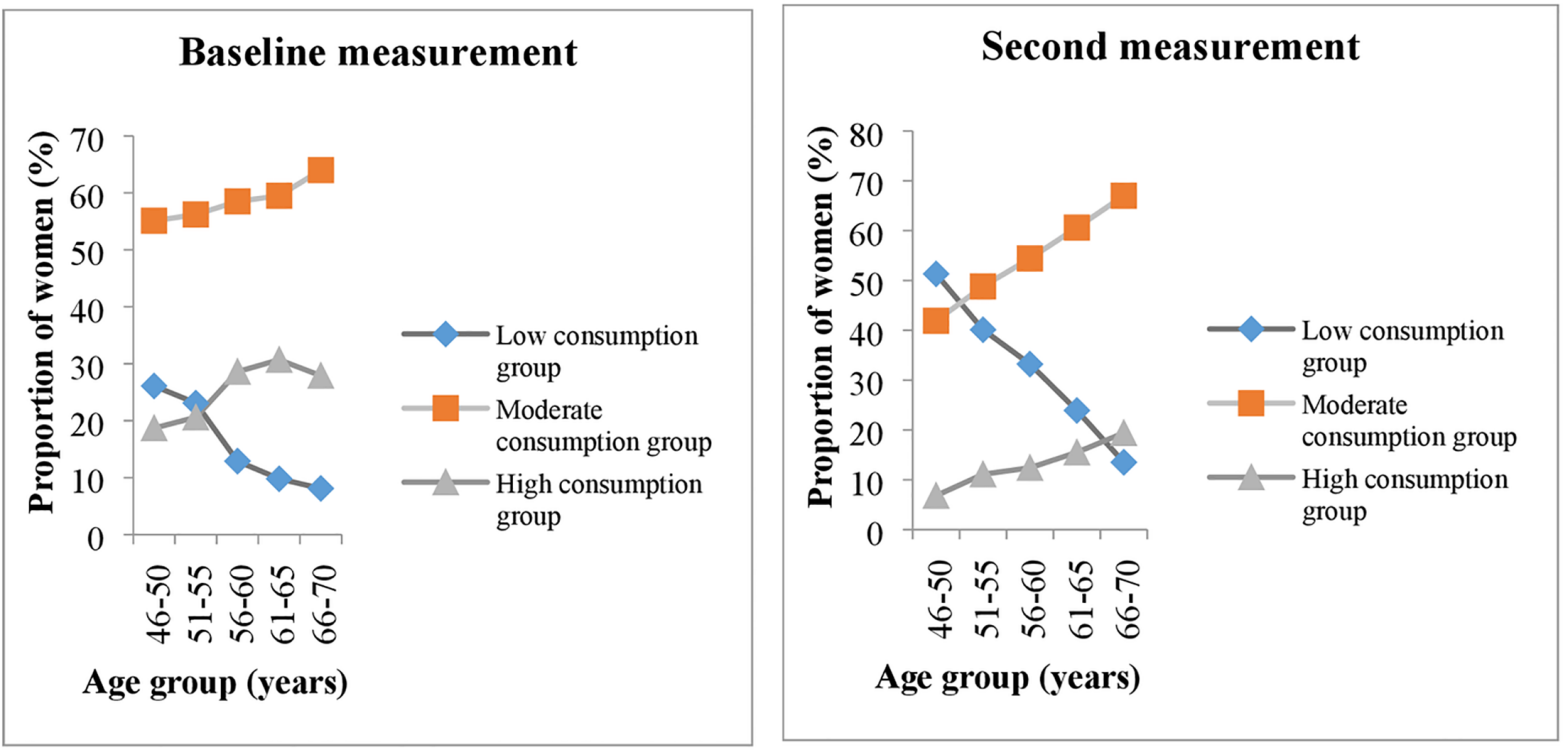
Pattern of potato consumption (%) by age group at baseline and second measurement respectively. (Low: <1 potato per day. Moderate: 1–2 potatoes per day. High: ≥ 3 potatoes per day).

With regards to consumption across age groups, a general pattern was observed at baseline and at second measurement. The percentage of women in the low consumption group was higher in the youngest age group and lower in the oldest while the percentage of women in the moderate and high consumption groups had the opposite tendency (Fig 3). This is consistent with the positive correlation (p < .001) observed between potato consumption and age group (Table 2). The shift towards a lower potato consumption group took place in all the age groups (Result not shown).

Our age-adjusted model showed that for region of residence, ‘living in the north’ compared to ‘living in Oslo’ made the strongest unique contribution to explaining potato intake at second measurement (B: 0.67, 95% CI: 0.62–0.72) (Table 4). The further north a participant lived at first measurement, the stronger the region of residence predicted the follow-up potato intake, and there was a clear pattern of increase in the intake from south to north. Though this association was weaker in the mutually adjusted model, the trend was still very clear (Table 5).

**Table 4:** Change in potato intake from baseline to second measurement in age-adjusted linear regression analysis by baseline characteristics. The NOWAC study^1^.

Lifestyle, socioeconomic and health-related variables	Change in potato consumption estimates^2^				
	B	95% CI for B		p-value	R^2^
Adjusted for potato intake at baseline^2^					
Age (years) (Ref. 40–49)					
50–59	.27	.24	.29	.00	.32
60–70	.56	.52	.60	.00	
Adjusted for age and potato intake at baseline					
Education(years) (Ref. ≤9)					
10–12	-.16	-.19	-.13	.00	
≥13	-.35	-.38	-.32	.00	.33
Income					
≤300,000	.01	-.02	.04	.57	
(ref. 301,000–450,000)					
451,000–600,000	-.16	-.20	-.13	.00	.33
>600,000	-.34	-.38	-.29	.00	
Household structure (Ref. Living with partner)					
Living alone	-.12	-.16	-.07	.00	.32
Living with children	.00	-.03	.03	.91	
Region of residence (Ref. Oslo)					
East (except Oslo)	.29	.25	.34	.00	
South	.38	.31	.45	.00	
West	.40	.35	.45	.00	.34
Mid	.39	.33	.45	.00	
North	.67	.62	.72	.00	
Smoking status (Ref. Never)					
Former	-.06	-.08	-.03	.00	.33
Current	.12	.09	.14	.00	
BMI (Ref. Normal/underweight)					
Overweight	.00	-.03	.03	.97	.32
Obese	.00	-.04	.05	.93	
Physical activity (Ref. Moderate)					
Low	.02	-.02	.05	.42	.32
High	.02	-.02	.05	.37	
Diabetes (Ref. Non-Diabetics)					
	-.02	-.12	.08	.73	.32

^1^n = 38,820. B = Unstandardised beta coefficient, p = significance value, CI: confidence interval, BMI: body mass index NOWAC: The Norwegian Women and Cancer Study

^2^The dependent variable was potato intake at second measurement. Intake at second measurement adjusted for baseline intake = change in potato intake

**Table 5:** Change in potato intake from baseline to second measurement in multivariable linear regression analysis by baseline characteristics. The NOWAC study^1^.

Lifestyle, socioeconomic and health-related variables	Change in potato consumption estimates^2^			
	B	95% CI for B		p-value
Age (years) (Ref. 40–49)				
50–59	.22	.19	.25	.00
60–70	.42	.37	.46	.00
Education (years) (Ref. ≤9 years)				
10–12	-.11	-.14	-.08	.00
>13	-.26	-.29	-.22	.00
Income				
≤300,000	-.00	-.03	.03	.10
(ref. 301,000–450,000)				
451,000–600,000	-.11	-.14	-.07	.00
>600,000	-.23	-.27	-.18	.00
Household structure (Ref. Living with partner)				
Living alone	-.13	-.17	-.09	.00
Living with children	.01	-.02	.04	.41
Region of residence (Ref. Oslo)				
East (except Oslo)	.23	.19	.28	.00
South	.31	.24	.37	.00
West	.33	.28	.38	.00
Mid	.32	.26	.38	.00
North	.60	.55	.65	.00
Smoking status (Ref. Never)				
Former	-.09	-.12	-.06	.00
Current	.03	.00	.06	.04
BMI (Ref. Under/normal)				
Overweight	-.04	-.06	-.01	.01
Obese	-.06	-.11	-.02	.01
Physical activity (Ref. Moderate)				
Low	.01	-.03	.05	.60
High	-.01	-.04	.03	.71
Diabetes (Ref. Non-Diabetics)				
	-.04	-.14	.06	.05
Potato intake at first measurement	.52	.51	.53	.00

^1^n = 38,820. B:Unstandardised beta coefficient, p: significance value, CI: confidence interval, BMI: body mass index, R^2^ = .347, NOWAC: The Norwegian Women and Cancer Study. All variables are mutually adjusted.

^2^The dependent variable was potato intake at second measurement. Intake at second measurement adjusted for baseline intake = change in potato intake

Living alone was associated with a lower intake of potato at second measurement compared to living with a partner (B: -0.12, 95% CI: -0.16 –-0.07) and living with children was associated with a statistically insignificantly higher intake at second measurement (Tables 4 and 5).

Although BMI showed no effect on potato intake in the age adjusted model, being overweight or obese at first measurement was associated with a statistically significant lower intake of potato at second measurement compared to being underweight/normal in the mutually adjusted model (B: -0.04, 95% CI: -0.06 –-0.01 and B: -0.06, 95% CI: -0.11 –- 0.02 respectively). The result for physical activity was insignificant in both the age-adjusted and mutually adjusted models (Tables 4 and 5).

Having completed secondary school or higher education was associated with consumption of less potato at follow-up. This association was statistically significant (p<0.001) in both the age-adjusted and mutually adjusted models. All income categories were associated with a lower intake of potato at second measurement compared to the reference group (301,000–450,000 NOK). The association seen in the income groups above the reference group were however stronger than that of the income group below the reference group. The trends were similar in both the age-adjusted and mutually-adjusted models but the association was weaker in the latter, and the lowest income group did not differ from the reference group after mutual adjustment (Tables 4 and 5). Baseline age also showed a positive linear association with follow-up potato intake (B: 0.27, 95% CI: 0.24–0.29 for age group 50–59 and B: 0.56, 95% CI: 0.52–0.60 for age group 60–70 compared to age group 40–49). The same pattern but a weaker association was observed in the mutually adjusted model. The baseline mean age of women in the low consumption group was 48.54 years while that of the moderate and high consumption groups were 50.82 years and 51.87 years respectively. Women who reported being former smokers at baseline consumed less potatoes at follow-up than never smokers (B: -0.06, 95% CI: -0.08 –-0.03), while those who reported being current smokers at baseline consumed more potatoes at follow-up than never smokers (B: 0.12, 95% CI: 0.09–0.14). After mutual adjustment, the associations became weaker for current and stronger for former smokers (Tables 4 and 5).

In the mutually adjusted model, diabetics consumed less potato at follow-up compared to the baseline non-diabetics (B: -0.04, 95% CI: -0.14–0.06). This association was insignificant in the age-adjusted model (Tables 4 and 5).

Our model explained 34.7% of the potato intake at second measurement among women in the NOWAC study. Baseline potato intake was the strongest predictor of intake at follow-up.

## Discussion

The aim of our study was to examine the change in potato consumption among participants of the NOWAC study and factors that predicted the change. Although the absolute level of potato intake was still high, our results show a reduction in the average daily consumption of potatoes from 112 g/day at baseline to 95 g/day at second measurement, about 15% from 1998–2005. A previous study has reported more than a 40% reduction in potato consumption in Norway between the period 1975 to 1995 [28]. It is likely that potato is being replaced with rice and pasta based on the result from another study within our cohort, which found that women who ate more rice and pasta had lower odds of high potato consumption, even after adjustment for energy intake [29]. This decline is not peculiar to Norway alone. Similar trends have been reported in Sweden [30] and Finland [31] with potato being replaced with rice and pasta. The proportion of women classified as high potato consumers in our study decreased substantially during this period while that of the low potato consumers increased. Cross tabulation shows that the low and moderate consumers at first measurement tend to remain so while a sizeable proportion of the high consumers migrated to the low or moderate consumption group at second measurement.

Potatoes are typically consumed steamed by participants in the NOWAC study. Frequency distribution of potato consumption at the third measurement showed that for the women that reported eating potatoes boiled, 49.6%, 45.8% and 4.6% of them were in the low, moderate and high consumption groups respectively. For the women that reported eating potatoes fried, 99.1% and 0.9% of them were in the low and moderate consumption groups respectively. There was none in the high consumption group. In another study within our cohort, 50% of the women ate boiled potatoes at least once a day, compared to 1% of the women who ate fried potatoes at least once a day [29]. In the other 9 European countries involved in the European Prospective Investigation into Cancer and Nutrition (EPIC) study, the percentage of participants who prepare potatoes fried was higher compared to Norway. But majority of the participants in these countries, including Norway, prepare potatoes boiled [32]. However, consumption surveys show that sale of fresh potatoes has more than halved since the 1970’s while consumption of processed potatoes, such as fries and chips, now account for more than half of the total potato turnover at the wholesale level [33]. Considering the health implications of frequent consumption of fried foods [34], the directorate of health is working to increase the consumption of potatoes at the expense of fatty potato products [33].

Our study showed that baseline region of residence was the best predictor of potato consumption at second measurement among women in the NOWAC study with a striking north-south gradient in the pattern of consumption. Living outside Oslo was associated with higher potato intake at second measurement. This is not surprising considering our findings from cross sectional analysis of NOWAC data which showed that potatoes are consumed more outside Oslo [29]. The further away from Oslo a woman lived at first measurement the higher the intake at second measurement. One possible explanation could be the fact that Oslo is the largest and most cosmopolitan city in Norway with the largest population of immigrants and thus subject to the influence of foreign foods and cultures [35]. The desire for these foreign foods could lead to a drop in the consumption of traditional foods like potatoes.

Our study showed a linear association between age and potato consumption with potato being consumed more by women in the older age groups. In addition, the mean age of women in the low consumption group was lower than the mean age of women in the high potato consumption group. Our study also showed that the largest reduction of the high potato consumption group from the first to the second measurement was in the age group 46–50 years. Possibly, women in this age group pay more attention to weight gain and are thus more likely to cut down on consumption of potatoes considering the long-term association of potato with weight gain [36]–older women may adhere more strongly to traditional diets.

This study showed that women in the higher income groups had lower potato intake at second measurement and this is consistent with earlier cross sectional research [28]. Other studies have reported that people of high SES consumed less traditional foods like potato and prefer modern healthy foods such as fruits and vegetables [37, 38]. They are also likely to be more aware of international trends as well as possess higher health literacy. A previous study reported that beyond cost considerations, factors like traditional food-related views, health-related education, and self-imposed discipline regarding lifestyle and diet shape the diet of people of low SES [39]. These factors could be an explanation for the high intake of potatoes among participants of low SES observed in our study.

Higher education was associated with a lower potato intake at the second measurement while lower education was associated with higher intake at second measurement. This finding is similar to that observed in a previous study [40]. Women with high education will most likely be in the high socioeconomic class and can thus afford a wide variety of food choices. They are also likely to have travelled and interacted more with people of other cultures, further increasing the variety of food at their disposal. Women with high education are quicker at picking international trends; the lower consumption observed among them could be a reflection of the international decline in potato consumption [28].

There was a lower intake of potato at second measurement among women living alone compared to women living with a partner. It is likely that single people choose to make easier, less time-consuming meals and probably eat convenience foods like pizza and hamburger [28]. Our result showed a higher intake of potato at second measurement among women living with children compared to women living with a partner. This is concordant with other studies [28]. A possible explanation could be the need to keep food budget low, and probably the fact that households with children sometimes opt for traditional meals in order to get children acquainted with such foods at an early age.

Non-diabetics had higher intake of potatoes at second measurement than diabetics. This is not surprising considering the fact that potatoes have a high glycemic index [8] and diabetics are advised to avoid such foods because they easily raise blood sugar [41]. The percentage of non-diabetics who migrated to the low potato consumption group at second measurement was higher than that of the diabetics. This may be part of the general decrease in potato consumption observed in this study.

Current smoking was associated with a higher intake of potato and former smoking was associated with a lower intake of potato at second measurement. This result is consistent with a previous cross sectional study which found that current smokers consumed French fries and some other foods like eggs and meat more frequently than former smokers and never smokers [42]. Quitting smoking is known to lead to weight gain [43]. Reducing the amount of potato consumed, and probably other foods, especially those that accelerate weight gain, may be a deliberate effort by former smokers to avoid excessive weight gain.

The main strength of this study is its large sample size and the fact that the sample is population-based and representative of Norwegian women 40–70 years although women in the NOWAC cohort are on average slightly more educated than the general female population. In addition, the external validity of the NOWAC study instruments has been found acceptable [20]. This is the first Norwegian study to examine the change in potato consumption in a cohort using the repeated measures design.

One limitation of this study is the fact that the questionnaires were self-administered and could have resulted in under-reporting or over-reporting of some values. The participants are randomly selected and it has been previously shown that they are representative of the female Norwegian population as a whole except for higher education than non-responders [20]. The prevalence of diabetes in the NOWAC cohort might seem low but is similar to that of the general population (2.3% in 2004) [44]. The cancer rates are the same in our cohort as in the general population of the same age [19] and the prevalence of obesity in this paper is slightly lower than that of the general population (10%, 2012) [45]. Also, as mentioned above, the diet questions have been validated, as has the question on physical activity [26], diabetes [21] and BMI [27], and the results are in the same range as those found in other cohorts. Also, only one question was asked regarding preparation methods of potatoes to the women in the NOWAC study, but as shown above, this is the preferred preparation method in this age group. The study was restricted to women. It has been reported that men consume more potatoes than women [46], but as noted earlier, the decline in potato consumption has been reported in the general population as well, so we expect to see similar trends among men in the same age group.

## Conclusion

The women in the NOWAC study reported a decrease in the intake of potato during the period studied but the absolute level of intake is still high. Non-dietary variables like diabetes, living alone, younger age, high SES, being a former smoker and being overweight or obese were associated with lower potato consumption at second measurement. Geography, aging, smoking, living with children and engaging in moderate or high level of physical activity were associated with higher potato intake at second measurement. The use of repeated measures design is necessary to understand the stability and direction of the possible change in diet of a population and will also give more precise estimates of the associations between potato consumption and human health.

## Abbreviations

B: Unstandardised beta coefficient
BMI: Body mass index
CI: confidence interval
FFQ: food frequency questionnaire
NOK: Norwegian Kroner
NOWAC: The Norwegian women and Cancer study
R^2^: Percentage of variance in outcome measure explained
SES: Socioeconomic status
